# Resolvin D1 and Resolvin E1 Promote the Resolution of Allergic Airway Inflammation via Shared and Distinct Molecular Counter-Regulatory Pathways

**DOI:** 10.3389/fimmu.2012.00390

**Published:** 2012-12-28

**Authors:** Bruce D. Levy

**Affiliations:** ^1^Pulmonary and Critical Care Medicine, Brigham and Women’s Hospital, Harvard Medical SchoolBoston, MA, USA

**Keywords:** resolution, resolvins, inflammation, lung, asthma

## Abstract

Resolvins are generated from omega-3 fatty acids during inflammatory responses in the lung. These natural mediators interact with specific receptors to decrease lung inflammation and promote its resolution in healthy tissues. There are several lung diseases of chronic inflammation that fail to resolve, most notable asthma. This common disorder has a lifetime prevalence of nearly 10% and is characterized, in part, by chronic, non-resolving inflammation of the airway. Pro-resolving mediators are generated during asthma; however, their biosynthesis is decreased in severe and uncontrolled asthma, suggesting that the chronic, adaptive inflammation in asthmatic airways may result from a resolution defect. This article focuses on recent insights into the cellular and molecular mechanisms for resolvins that limit adaptive immune responses in healthy airways.

## Introduction

The recent identification of specialized mediators that promote tissue resolution from acute inflammation and injury has opened a new window for discovery of cellular and molecular mechanisms governing chronic inflammation and adaptive immunity. Chronic “unresolved” inflammation is a pathologic response that is associated with several common human diseases for which there is no cure. Asthma is an exemplary illness with chronic inflammation that has a lifetime prevalence of nearly 1 in 10 in Western countries (Fanta, 2009). The chronic inflammation in asthma consists of airway infiltration of eosinophils and T-lymphocytes with increased levels of pro-phlogistic cytokines and lipid mediators (Busse and Lemanske, 2001). Of interest, many patients with uncontrolled lung inflammation, including clinically severe asthma, display a defect in the generation of specialized pro-resolving mediators (Levy et al., 2005, 2007; Planaguma et al., 2008; Table 1), consistent with a failure to establish sufficient protective counter-regulatory pathways in the asthmatic lung.

**Table 1:** **Uncontrolled lung inflammation – A defect in specialized pro-resolving mediators**.

Pro-resolving mediator	Disease	Finding	Reference
Lipoxin A_4_	Aspirin-exacerbated respiratory disease	Aspirin-tolerant asthmatics generate more lipoxins than aspirin-intolerant asthmatics	Sanak et al. (2000), Celik et al. (2007), Yamaguchi et al. (2011)
	Severe asthma	Diminished lipoxin biosynthesis in severe asthma	Levy et al. (2005), Vachier et al. (2005), Celik et al. (2007), Planaguma et al. (2008), Bhavsar et al. (2010), Wu et al. (2010), Fritscher et al. (2012)
	Bronchoconstriction in asthma	Protects against bronchoprovocation by either LTC_4_ or exercise	Tahan et al. (2008), Christie et al. (1992)
	Asthma exacerbation	Decreased lipoxin levels in exhaled breath during exacerbation	Hasan et al. (2012)
	Cystic fibrosis	Decreased generation and actions in cystic fibrosis	Karp et al. (2004), Yang et al. (2012), Chiron et al. (2008), Mattoscio et al. (2010)
	Scleroderma lung disease	Decreased lipoxin levels in BALFs	Kowal-Bielecka et al. (2005)
Resolvin E1	Cystic fibrosis	Decreased resolvin E1 levels in cystic fibrosis with a relationship to lung function	Yang et al. (2012)
Protectin D1	Asthma exacerbation	Decreased Protectin D1 in uncontrolled asthma	Levy et al. (2007)

Catabasis is a healthy host tissue response to noxious stimuli. The literal definition of catabasis refers to a military retreat and the term has been adopted for use to describe the resolution process of returning an inflamed or injured tissue to homeostasis after the “battle” of inflammation. Catabasis requires well orchestrated cellular responses in which soluble mediators appear to play critical roles (Serhan, 2007). For resolution (Majno, 1996), restitution of endothelial and epithelial cell barrier integrity is necessary to prevent continued edema formation. Additional granulocyte recruitment is blocked and those granulocytes that have infiltrated the tissue undergo programmed cell death. The apoptotic cells are then cleared, principally by macrophages. The phagocytes also clear tissue microbes and debris, and structural cells re-establish organ function. One family of mediators for these pro-resolving cellular actions is the resolvins, which are enzymatically derived from the omega-3 fatty acids eicosapentaenoic acid (i.e., E-series resolvins) and docosahexaenoic acid (i.e., D-series resolvins; Serhan et al., 2000, 2002). Together with the lipoxins, protectins, and maresins, the resolvins comprise a new genus of endogenous, specialized pro-resolving mediators (Serhan, 2007). These mediators serve as agonists at select receptors to transduce their cell type specific pro-resolving actions (Serhan, 2007). Defects in resolvin signaling pathways can be resolution “toxic” in model systems, leading to conversion of acute inflammatory responses to more chronic pathologic inflammation (Schwab et al., 2007), supporting a potential link for defective resolvin signaling to chronic inflammatory diseases. Of interest, there appears to be population heterogeneity in resolution mechanisms. Randomly selected healthy individuals display significant differences in the pace of resolution for acute exudative inflammation with segregation of these apparently healthy subjects into discrete cohorts of rapid and delayed resolvers (Morris et al., 2010) that may be partially explained by genetic variability, which has recently been linked to inflammatory disease (Simiele et al., 2011). In health, the conversion of acute to chronic airway inflammation is prevented by endogenous pro-resolving mechanisms for tissue catabasis.

Intrinsically linked to innate immune responses, the development of adaptive immunity is essential to host defense, but unregulated adaptive inflammation can also lead to disease, including autoimmune disorders, transplant rejection, and allergy. As part of a series of review articles exploring the research theme of “Resolution of inflammation: leukocytes and molecular pathways as potential therapeutic targets,” this article will focus on the regulation of adaptive inflammatory responses by resolvins, in particular shared and distinct counter-regulatory mechanisms for resolvin E1 and resolvin D1 in allergic inflammation.

## Resolvins and Their Receptors in the Lung

Eicosapentaenoic acid is an essential fatty acid that can be enzymatically converted to E-series resolvins, including resolvin E1, resolvin E2, and resolvin E3, during inflammation in mammals and fish (Serhan et al., 2000; Isobe et al., 2012). These mediators display stereoselective and cell type specific actions. Resolvin E1 (5*S*,12*R*,18*R*-trihydroxy-6*Z*,8*E*,10*E*,14*Z*,16*E*-eicosapentaenoic acid) can transduce its biological actions by interacting with specific G-protein coupled receptors, namely chemokine-like receptor 1 (CMKLR1) and leukotriene B_4_ receptor 1 (BLT1; Arita et al., 2005a, 2007). RvE1 serves as an agonist for CMKLR1, which is expressed in macrophages, dendritic cells (DCs), natural killer (NK) cells, and other T cells, and RvE1 serves as a receptor antagonist at BLT1 for leukotriene B_4_. BLT1 is expressed on granulocytes, T cells, and macrophages. Resolvin E1 and resolvin E2 (5*S*, 18*R*-dihydroxy-6*E*,8*Z*,11*Z*,14*Z*,16*E*-eicosapentaenoic acid) are generated via the actions of 5-lipoxygenase (ALOX5) from a common precursor 18-hydroxyeicosapentaenoic acid (18-HEPE) with two parallel stereospecific pathways (Arita et al., 2005a; Tjonahen et al., 2006; Oh et al., 2012). Resolvin E3 [17,18(*R*/*S*)-dihydroxy-5*Z*,8*Z*,11*Z*,13*E*,15*E*-eicosapentaenoic acid] is distinct from RvE1 and RvE2 because it is generated via the actions of 12/15-lipoxygenase (ALOX12/15; Isobe et al., 2012). Since ALOX5 and ALOX12/15 are generally compartmentalized into distinct leukocyte classes, neutrophils (ALOX5) appear to play significant roles in RvE1 and RvE2 generation, while eosinophils (ALOX12/15) are significant in RvE3 biosynthesis.

Docosahexaenoic acid is another essential omega-3 fatty acid that can be enzymatically converted to resolvins. In a lipoxygenase-dependent manner, DHA is transformed to D-series resolvins, including resolvin D1–D6, during inflammation (Serhan, 2007). These mediators also display stereoselective and cell type specific actions. Resolvin D1 (7*S*,8*R*,17*S*-trihydroxy-4*Z*,9*E*,11*E*,13*Z*,15*E*,19*Z*-docosahexaenoic acid) transduces its biological actions by interacting with specific G-protein coupled receptors, including the lipoxin A_4_ receptor ALX/FPR2 and, in humans, GPR32 (Sun et al., 2007; Krishnamoorthy et al., 2010, 2012). RvD1 serves as an agonist for both of these receptors. ALX/FPR2 is broadly expressed in many cells types, including most leukocytes as well as structural cells, such as airway epithelial cells (Chiang et al., 2006). GPR32 is expressed on phagocytes (Krishnamoorthy et al., 2010). There are also aspirin-triggered 17*R* D-series resolvins (AT-RvD1–4) that are generated in human and murine tissues, including lung, and AT-RvD1 can also interact with ALX/FPR2 and GPR32 receptors (Krishnamoorthy et al., 2012). Of note, in an aspirin-independent manner, cytochrome P450 enzymes, which are abundant in the lung, can also convert DHA to 17*R*-hydroxy-DHA that can serve as a precursor for 17*R*-RvD1 (i.e., AT-RvD1), so the presence of aspirin is not required for AT-RvD1 generation.

Relatively little information is currently available on most of these recently identified E-series and D-series resolvins regarding their actions during lung inflammation; however, recent evidence has identified important roles for resolvin E1 and resolvin D1 and their cellular targets in promoting the resolution of lung inflammation (Aoki et al., 2008; Haworth et al., 2008, 2011; Seki et al., 2010; Bilal et al., 2011; Wang et al., 2011; Eickmeier et al., 2012; El Kebir et al., 2012; Rogerio et al., 2012), including allergic airway responses (Aoki et al., 2008; Haworth et al., 2008, 2011; Bilal et al., 2011; Rogerio et al., 2012).

## Expression of CMKLR1 – Cell Type, Lung Tissue, Asthma

CMKLR1 is highly expressed in immature plasmacytoid DCs and at lower levels in myeloid DCs, macrophages, and NK cells (Arita et al., 2005a; Parolini et al., 2007). The CMKLR1 signaling pathway is structurally and functionally conserved between human and mouse. In a model of zymosan induced peritonitis, CMKLR1 deficient mice, exhibit increased inflammation, indicating that this receptor is important for counter-regulatory signaling (Cash et al., 2008). Both RvE1 and chemerin can interact with CMKLR1 and display potent anti-inflammatory properties in LPS-induced acute lung inflammation in mice, reducing neutrophil infiltration and inflammatory cytokine release in a CMKLR1-dependent manner (Luangsay et al., 2009).

The expression of CMKLR1 in plasmacytoid DCs suggests an important role in anti-viral immunity. When wild-type and CMKLR1 knock-out mice are infected by pneumonia virus of mice, the CMKLR1 deficient mice display higher mortality and morbidity, alteration of lung function, delayed viral clearance and increased neutrophilic infiltration. The CMKLR1 deficient mice have a lower recruitment of plasmacytoid DCs and a reduction in type I interferon production. Recruitment of plasmacytoid DCs via CMKLR1 contributes to adaptive immune responses and viral clearance, but also enhances the inflammatory response. Anti-inflammatory pathways involving CMKLR1 expressed by non-leukocytic cells in the lung also contribute to the increased morbidity/mortality in CMKLR1 deficient mice (Bondue et al., 2011).

Of interest, CMKLR1 signaling in acute lung inflammation appears context dependent. In a separate model of acute lung inflammation, cigarette smoke-induced lung inflammation was attenuated in CMKLR1 deficient mice with decreased levels of inflammatory chemokines and inflammatory cells. In addition, the infiltration of leukocytes persists for 14 days after cessation of smoke exposure in this model in wild-type mice, but the CMKLR1 deficient mice have a marked decrease in lung T cells at this time point (Demoor et al., 2011). Together, these findings indicate that the RvE1 receptor CMKLR1 is expressed in the lung by both leukocytes and structural cells and CMLKR1 signaling plays pivotal roles in the regulation of innate and adaptive immune cell activation in the lung.

The recruitment of CMKLR1-expressing leukocytes to the lung is regulated during inflammatory responses. Following acute LPS-induced lung inflammation, NK cells expressing CMKLR1 are recruited to the airways in a CCRL2-dependent manner. Engagement of CCRL2 on endothelial cells initiates adhesion of CMKLR1-expressing lymphoid cells through an α(4)β(1) integrin/VCAM-1-dependent mechanism. CCRL2 expression by endothelial cells is regulated by cell activation, so CMKLR1-dependent lymphocyte adhesion to endothelial cells can be targeted to sites of inflammation, including inflamed lung (Monnier et al., 2012).

## Expression of ALX/FPR2 – Cell Type, Lung Tissue, Asthma

ALX/FPR2 is expressed in several types of leukocytes (Chiang et al., 2006), including neutrophils (Fiore et al., 1992, 1994), monocytes (Maddox and Serhan, 1996), eosinophils (Levy et al., 2002), myeloid progenitors (Stenke et al., 1991), NK cells (Ramstedt et al., 1987; Haworth et al., 2011), and activated T cells (Ariel et al., 2003), as well as resident cells such as macrophages (Godson et al., 2000), synovial fibroblasts (Sodin-Semrl et al., 2000), and intestinal epithelial cells (Gronert et al., 1998). ALX/FPR2 is expressed in murine and human lung (Planaguma et al., 2008; Rogerio et al., 2012), airway epithelial cells (Bonnans et al., 2003, 2006), and alveolar macrophages (Rogerio et al., 2012). As early as 2 h after acute lung injury or inflammation, ALX/FPR2 expression increases in mucosal epithelial cells (Bonnans et al., 2006). Counter-regulatory signaling via ALX/FPR2 has been demonstrated *in vivo* using ALX/FPR2 deficient mice (Dufton et al., 2010) and transgenic mice that express human ALX/FPR2 directed by a component of the myeloid CD11b promoter (Devchand et al., 2003). ALX/FPR2 deficient mice have more marked inflammatory responses with increased leukocyte adherence and emigration into inflamed tissue after ischemia-reperfusion injury and after carrageenan-induced paw edema. In addition, ALX/FPR2 knock-out mice display increased sensitivity to arthrogenic serum and fail to resolve from this chronic inflammatory arthritis (Dufton et al., 2010). Also of note, human ALX/FPR2-transgenic mice have decreased inflammatory responses and are protected from the development of allergic airway inflammation with markedly decreased eosinophil activation and tissue accumulation (Levy et al., 2002). In asthma, ALX/FPR2 receptor expression is regulated in a cell type specific manner with decreases in peripheral blood neutrophil and eosinophil expression in this chronic inflammatory condition (Planaguma et al., 2008).

Recently, in subjects with chronic obstructive pulmonary disease, serum amyloid A (SAA) was identified as a biomarker for acute exacerbations (Bozinovski et al., 2008). SAA can also interact with ALX/FPR2 receptors, and unlike RvD1 or LXA_4_, the SAA-ALX/FPR2 interactions are pro-inflammatory (Bozinovski et al., 2012). Because plasma levels of SAA are at least two-log orders higher than LXA_4_ during acute exacerbations (Bozinovski et al., 2012), the pro-inflammatory SAA-ALX/FPR2 signaling can overwhelm the pro-resolving mediator protective signaling at this receptor. The balance of ALX/FPR2 ligands during asthma and the influence of corticosteroids is a subject of on-going investigation.

## Allergic Airway Responses – An Experimental Model of Adaptive Immunity and Asthma

Animal models have not been developed that fully resemble human asthma, but they are quite useful for investigation of adaptive immunity and asthma traits. To model allergic airway inflammation, animals are first sensitized to an allergen and then challenged by respiratory tract exposure to the same allergen (Kips et al., 2003; Corry and Irvin, 2006; Pichavant et al., 2007; Zosky and Sly, 2007). Roles for representative family members of D-series resolvins and E-series resolvins have been determined using a model in which chicken ovalbumin (OVA) serves as an allergen for in-bred mice. The animals are sensitized by intraperitoneal injection of OVA combined with the adjuvant aluminum hydroxide to initiate a strong Th2 phenotype (Aoki et al., 2008; Haworth et al., 2008, 2011; Bilal et al., 2011; Rogerio et al., 2012). In sensitized mice, OVA aerosol challenge on four consecutive days leads to adaptive inflammation consisting of predominantly eosinophils and T-lymphocytes, in particular in medium to small airways and alveoli (Levy, 2010). There is also perivascular inflammation. Antigen-induced responses also increase airway mucus metaplasia and hyper-responsiveness (Levy, 2010). To determine the extent of the airway hyper-responsiveness, methacholine is administered via inhalation while the mice are intubated and sedated on a ventilator circuit. A dose response curve is constructed for methacholine-initiated changes in lung resistance.

In most instances, the airway inflammation of asthma in humans does not resolve completely; however, in healthy airways, inhalation of potential allergens or provocative stimuli leads to an acute inflammatory response that is self-limited. Several classes of natural anti-inflammatory mediators, including resolvins, have been identified in inflamed airways (Bilal et al., 2011; Eickmeier et al., 2012). Because the clinical presentation of asthma is after the disease has already developed, more recent research has focused on the natural factors that promote resolution of allergic airway responses and identification of potential disease mechanisms that counter these endogenous, protective signals to perpetuate inflammation and potentially maladaptive airway responses. In the murine model of allergic airway responses described above, the cessation of OVA aerosol challenge leads to self-limited lung inflammation with resolution of the adaptive immune responses within 1–2 weeks (Haworth et al., 2008). Investigation of the resolution phase of allergic airway responses has uncovered several pro-resolving molecular and cellular mechanisms for adaptive airway inflammation (Levy et al., 2007; Haworth et al., 2008, 2011; Rogerio et al., 2012). NK cells were recently assigned important roles for clearance of antigen-specific T cells. During the natural resolution phase of allergic airway inflammation, eosinophils, and T cells decrease markedly concomitant with an increase in the numbers of NK cells in the lung and associated mediastinal lymph nodes (Haworth et al., 2011). These resolution NK cells also acquire cell surface markers, including NKG2D, consistent with NK cell activation (Haworth et al., 2011). The timely resolution of allergic airway inflammation is prolonged when NK cells are depleted, blocked from interacting with target cells, or inhibited from migrating to the lung, leading to a persistence of airway eosinophils and antigen-specific CD4 (Haworth et al., 2011) T cells (Haworth et al., 2011). Lung macrophages also serve important pro-resolving roles in the clearance of allergic airway inflammation. After the respiratory tract is exposed to allergen, lung macrophages display a significant capacity for clearance of airway antigen that was introduced during aerosol challenge (Rogerio et al., 2012). These recent findings identify important pro-resolving roles for innate lung tissue lymphocytes and macrophages in the regulation of adaptive immune responses.

## Actions of RvD1 and RvE1 in Allergic Airway Inflammation

LC-MS/MS-based lipido-metabolomic analyses of inflamed murine lung reveals picogram quantities of RvD1 and RvE1 that can be increased several fold with increased substrate availability (Bilal et al., 2011; Eickmeier et al., 2012). In asthma, airway mucosal epithelial cells have depleted stores of docosahexaenoic acid (Freedman et al., 2004) and lower levels of 17-hydroxy-DHA and protectin D1 in exhaled breath condensates compared with healthy control subjects (Levy et al., 2007). Recently, the potential beneficial actions of resolvins in experimental models of airway mucosal inflammation have been reported.

Lung expression of the RvD1 receptor ALX/FPR2 is induced *in vivo* in allergic airway inflammation (Levy et al., 2002). When RvD1 is given to OVA-sensitized mice just prior to OVA aerosol challenge, the development of allergic airway responses is significantly decreased (Rogerio et al., 2012). In particular, RvD1 markedly decreases eosinophils and levels of IL-4, IL-5, and IL-13 in bronchoalveolar lavage fluids (BALFs), consistent with a dominant effect of the mediator on the development of Th2 adaptive inflammation. Regulation of these cytokines by RvD1 is associated with a significant increase in lung IκBα, suggesting decreased activation of NF-κB. Airway mucus metaplasia is also decreased by RvD1 with a more modest effect on airway hyper-responsiveness to methacholine. BALF levels of the counter-regulatory mediators IL-10 and LXA_4_ are not increased by RvD1 administration, indicating non-redundant anti-inflammatory signaling circuits for these mediators.

Potent regulation by RvD1 and AT-RvD1 of the allergen-driven accumulation of eosinophils was linked to significant decrements in BALF IL-5, eotaxin, and LTB_4_ (Rogerio et al., 2012). In many asthmatics, a Th2 cytokine gene expression signature is induced (Woodruff et al., 2007), including IL-5, which is an important cytokine for the recruitment and activation of eosinophils, especially in conjunction with eotaxins (Busse and Lemanske, 2001). IL-5, eotaxin, NF-κB activation (Yang et al., 1998), and LTB_4_ (Terawaki et al., 2005) can all increase airway eosinophilia. In murine allergic airway inflammation, RvD1 and AT-RvD1 decreased each of these mediators of eosinophil activation and accumulation. Eosinophils may also play important roles in airway remodeling and can generate the pro-fibrotic growth factor TGF-β1 (Wong et al., 1991). The RvD1 and AT-RvD1 mediated decrease in eosinophils and TGF-β levels (Rogerio et al., 2012) suggest additional beneficial actions for these mediators in preventing chronic airway remodeling. Further study in models of chronic inflammation is needed to address this potential tissue protective role for these D-series resolvins.

When administered after acute airway inflammation is established, RvD1 significantly and rapidly decreases the allergic lung inflammation within 1 h (Rogerio et al., 2012). Because RvD1 is subject to rapid inactivation in the lung, it is impact is transient (Rogerio et al., 2012), so when RvD1 is given daily for three consecutive days, there is only a modest decrease in the BALF eosinophil resolution interval over the subsequent week (Rogerio et al., 2012). Of note, an equivalent dose and administration of AT-RvD1 (∼0.005 mg/kg) provides significantly greater pro-resolving actions than RvD1, including a marked decrease in the BALF eosinophil resolution interval by more than 50% – an approximate doubling of the pace of resolution! Both of these D-series resolvins are agonists at ALX/FPR2 receptors albeit with different binding kinetics (Perretti et al., 2002; Krishnamoorthy et al., 2010, 2012; Norling et al., 2012). In addition, the metabolism of these epimers is distinct (Sun et al., 2007). In the presence of lung macrophages, AT-RvD1 has a decreased rate of metabolic inactivation relative to RvD1 (Rogerio et al., 2012). RvD1 and AT-RvD1 are diastereomers, differing only in stereochemistry at carbon 17 (reviewed in Serhan et al., 2008). This change in stereochemistry for AT-RvD1 provides a significant increase in the mediator’s half-life *in vivo*, secondary to resistance to metabolic inactivation by eicosanoid oxidoreductases (Sun et al., 2007; Krishnamoorthy et al., 2012).

AT-RvD1 displays potent pro-resolving actions on molecular and cellular inflammatory responses. During resolution of allergic airway responses, BALF levels of IL-17, eotaxin, TARC, TGF-β, and LTB_4_ are significantly decreased by AT-RvD1 administration (Rogerio et al., 2012). For tissue catabasis after antigen challenge, it is essential to clear the allergen from the lung. Lung macrophages play critical roles in this catabatic process (Thornton et al., 2012). AT-RvD1 increases the macrophage phagocytosis index for OVA *in vitro* and *in vivo* (Rogerio et al., 2012). By promoting more rapid allergen clearance by lung macrophages, AT-RvD1 accelerates the pace of resolution of allergic airway responses, namely adaptive airway inflammation, mucus metaplasia, and hyper-responsiveness to methacholine.

Resolvin E1 is also a potent anti-inflammatory and pro-resolving mediator for allergic airway responses. Similar to RvD1, RvE1 can prevent the development allergic airway responses in this murine model of asthma (Levy et al., 2002, 2007; Haworth et al., 2008). When RvE1 is administered intravenously, it potently inhibits the induction of allergic airway inflammation (Aoki et al., 2008; Haworth et al., 2008) and when given during the resolution phase of inflammation, RvE1 accelerates the clearance of airway inflammation, mucus metaplasia, and hyper-reactivity to methacholine (Haworth et al., 2008, 2011). These RvE1-mediated bronchoprotective actions during resolution are multi-pronged, including inhibition of Th17 effector lymphocytes, engagement of activated NK cells and increased generation of interferon-gamma (IFN-γ) and LXA_4_ (Haworth et al., 2008, 2011). After cessation of allergen exposure, the timely resolution of allergic airway responses is governed by regulation of the Th17 pathway (Haworth et al., 2008). This is in sharp contrast to the pivotal roles for Th2 cytokines during the development of allergic lung inflammation (*vide supra*). IL-17 can be generated by several cell types in asthmatic lung, including Th17 cells that are a subset of CD4^+^ T helper cells characterized by the production of IL-17 and whose population expansion and survival depends upon IL-23. IL-17 has been linked to the pathogenesis of many inflammatory diseases, is present in the airways of asthmatic patients and can induce lung inflammation, airway hyper-reactivity and mucus production (Chen et al., 2003; Haworth et al., 2008; Al-Ramli et al., 2009; Alcorn et al., 2010; Lajoie et al., 2010). Similar to RvD1 and AT-RvD1, RvE1 also regulates IL-17 to promote resolution of allergic airways responses.

Identification of an important role for IL-17 in the persistence of lung inflammation, in particular during allergic airway inflammation, is supported by several lines of evidence. Transgenic expression of IL-17 in airway epithelial cells induces airway eosinophil and lymphocyte infiltration and structural changes with mucus metaplasia (Park et al., 2005). Mice deficient for the IL-17 receptor are protected from allergen-induced airway inflammation (Schnyder-Candrian et al., 2006), and in humans, asthmatic subjects have higher levels of BALF and sputum IL-17 (Molet et al., 2001; Barczyk et al., 2003; Schnyder-Candrian et al., 2006). Indicative of its importance in the lung, IL-17 may have dual roles in the regulation of allergic airway inflammation, as it can also inhibit T_H_2 immune responses in model systems (Schnyder-Candrian et al., 2006). In promoting resolution, RvE1 significantly decreases both IL-17 and allergic airway responses (Haworth et al., 2008), supporting a relationship between IL-17 and persistent airway inflammation.

In addition to IL-17, BALF levels of IL-23 are also decreased by administration of RvE1. IL-23 is also involved in the pathobiology of chronic inflammatory diseases, including colitis, encephalitis, psoriasis, rheumatoid arthritis, and cancer (Langrish et al., 2005; Chan et al., 2006; Langowski et al., 2006; Yago et al., 2007), and IL-23 is critical for the survival of T_H_17 cells (Langrish et al., 2005; Bettelli et al., 2006). IL-23 induces the release of pro-inflammatory chemokines from eosinophils, which express the IL-23 receptor (Cheung et al., 2008). Moreover, IL-23 deficient mice are protected in models of chronic inflammation, such as colitis (Yen et al., 2006). Of note, RvE1 also protects against the development of colitis in model systems (Arita et al., 2005b; Hudert et al., 2006). Thus, regulation of IL-23 by RvE1 appears critical to its pro-resolving actions in mucosal inflammation.

As mentioned above, NK cells express the RvE1 receptor CMKLR1 (Arita et al., 2005a; Parolini et al., 2007). NK cell depletion impairs RvE1’s protective actions for the timely resolution of adaptive inflammation (Haworth et al., 2011). RvE1 regulates NK cell homing, increases clearance of antigen-specific CD4^+^ T cells and increases NK cell cytotoxicity. Circulating and lung NK cell numbers increase with RvE1 administration, consistent with a role for RvE1 in NK cell transit through the inflamed lung to its associated mediastinal lymph nodes. RvE1 induces CXCL9 expression in the lung and mediastinal lymph nodes. Antibody-mediated inhibition of CXCL9-CXCR3 interactions blocks NK cell infiltration into inflamed lung and lymph nodes, and delays resolution of adaptive airway inflammation. These findings indicate that RvE1 can regulate tissue chemokines to target NK cell homing to the lung for catabasis. The influence of D-series resolvins and ALX/FPR2 signaling on NK cell functional responses is an area of active investigation.

Current anti-inflammatory therapeutic strategies for asthma include corticosteroids, CysLT1 receptor antagonists and anti-IgE antibody (Fanta, 2009). While little is known regarding the influence of these approaches on the actions of resolvins, their independent signaling pathways suggest that the resolvins would complement existing therapeutics. Corticosteroids share the resolvins’ anti-inflammatory actions on eosinophils and T cells; however, they do not share resolvins’ pro-resolving actions for macrophages and NK cells. CysLT1 receptor antagonists and anti-IgE antibody target specific pro-phlogistic pathways and would not be expected to interfere with the resolvins’ mechanisms of action. Regarding pharmacological considerations for the resolvins, these mediators carry potent actions in sub-nanomolar concentrations *in vitro* (reviewed in Serhan, 2007) and at doses of ∼0.005 mg/kg *in vivo* (Haworth et al., 2008; Rogerio et al., 2012). As with the Fat-1 transgenic mouse (Bilal et al., 2011), increasing the tissue levels of omega-3 fatty acids can increase resolvin formation and offer protection from allergic airway responses; however, dietary supplementation is less potent than direct administration of resolvins (Seki et al., 2010).

## Shared and Distinct Pro-Resolving Mechanisms for RvE1 and RvD1/AT-RvD1 in Adaptive Inflammation

**Figure 1:**
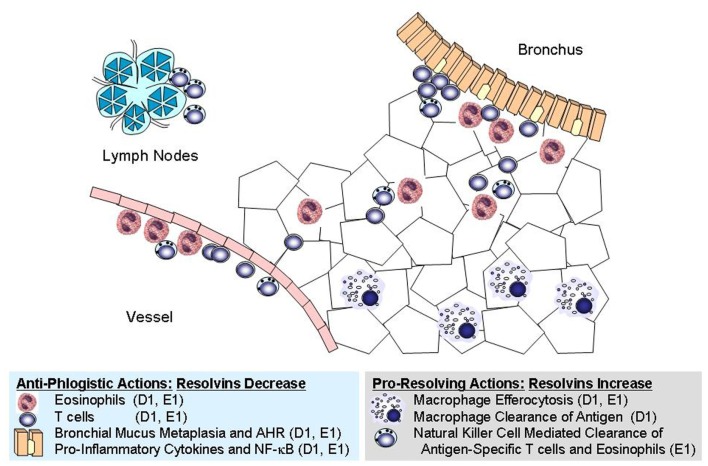
**Cellular targets for resolvins in allergic inflammation**. Shared and distinct anti-phlogistic and pro-resolving actions are illustrated for RvD1 (D1) and RvE1 (E1). These natural mediators have independent signaling pathways initiated via interactions with distinct receptors, yet they share many overlapping properties at the cellular and tissue levels.

RvE1 and RvD1/AT-RvD1 are agonists at distinct pro-resolving receptors, yet share many similar properties in the regulation of adaptive inflammation in the lung (Figure 1, Table 2). These mediators decrease recruitment of lung eosinophils, lymphocytes, and macrophages during adaptive immune responses and decrease allergic airway responses, including mucus metaplasia and hyper-responsiveness to methacholine. In addition, these specialized pro-resolving mediators share many similarities in the regulation of lung inflammatory peptide and lipid mediators. Despite these commonalities, the RvE1 and RvD1/AT-RvD1 pro-resolving pathways are not entirely redundant. In addition to their distinct receptors, there are clear differences between RvE1 and RvD1/AT-RvD1 in the regulation of BALF levels of IL-5, IFN-γ, and LXA_4_ during resolution (Haworth et al., 2008, 2011; Rogerio et al., 2012). While there is no data on concomitant administration of RvE1 and RvD1 in this model, co-administration of RvE1 and a bioactive LXA_4_ stable analog, which, like RvD1/AT-RvD1, interacts with ALX/FPR2 receptors, provides additive pro-resolving actions, yet there are important differences in their mechanisms. Both RvE1 and the LXA_4_ analog decrease BALF levels of IL-17, but distinct from RvE1, the LXA_4_ analog does not inhibit IL-23 production or increase IFN-γ levels (Haworth et al., 2008). These findings of shared and distinct points of counter-regulation for RvE1 and the LXA_4_ stable analog indicate the presence of independent pro-resolving signaling circuits, likely mediated by CMKLR1 and ALX/FPR2 respectively, which in this model of adaptive immunity converge on the regulation of IL-17 to promote catabasis (Table 2). Of interest, when administered during the upstroke of allergic inflammation, both RvD1 and RvE1 are also potent regulators of the development of airway hyper-responsiveness to methacholine, mucus metaplasia, eosinophil accumulation, and T_H_2 cytokine mediator release (e.g., IL-13; Haworth et al., 2008, 2011). Regulation of IL-13 by resolvins during induction of adaptive inflammation is distinct from their actions when given during resolution. When given after the adaptive inflammation is already established, these resolvins do not lead to significant changes in BALF levels of IL-13; however, both RvD1 and RvE1 lead to marked decreases in BALF levels of IL-17. While the mechanisms that induce adaptive inflammation (i.e., T_H_2 cytokines) are distinct from those linked to persistent mucosal inflammation (i.e., IL-17 and IL-23), the protective actions of resolvins include regulation of both of these important families of inflammatory mediators. Inhibition of IL-17 production appears to be a common point of regulation for RvE1 and RvD1 for resolution of allergic airway responses (Serhan et al., 2000; Haworth et al., 2011).

**Table 2:** **Shared and distinct pro-resolving mechanisms for RvE1 and RvD1/AT-RvD1 during murine adaptive inflammation**.

Receptors	RvE1	RvD1/AT-RvD1	LXA_4_ analog
	CMKLR1 BLT1	ALX/FPR2	ALX/FPR2 CysLT1
**ALLERGIC AIRWAY RESPONSES**			
Inflammation			
Eosinophils	Decreased	Decreased	Decreased
Lymphocytes	Decreased	Decreased	Decreased
NK Cells	Increased		
Macrophages	Decreased	Increased	
Mucus metaplasia	Decreased	Decreased	Decreased
MCh ED200	Increased	Increased	Increased
**CYTOKINES**			
IL-4	No change	No change	
IL-5	No change	Decreased	
IL-6	Decreased		
IL-10	No change	No change	
IL-13	No change	No change	
IL-17	Decreased	Decreased	Decreased
IL-23	Decreased	Decreased	No change
IL-27	Decreased		
Interferon-gamma	Increased	No change	Decreased
**CHEMOKINES**			
Eotaxin		Decreased	
TARC		Decreased	
**LIPID MEDIATORS**			
LTB_4_	Decreased	Decreased	
CysLTs	No change		
LXA_4_	Increased	No change	

## Summary and Conclusions

The recent discovery of resolvins, endogenously generated from the essential fatty acids docosahexaenoic acid and eicosapentaenoic acid, has uncovered molecular and cellular mechanisms for the resolution of acute and adaptive inflammation. These specialized and stereospecific pro-resolving chemical mediators play important roles in limiting allergic airway responses and promoting catabasis of the inflamed lung. In the lung, resolvins are enzymatically generated during cell–cell interactions, often between leukocytes and structural cells. Their interactions with specific receptors establish resolution circuits with cell type specific functional responses. While RvD1 and RvE1 share some cellular targets and pro-resolving actions, there is accumulating evidence for distinct counter-regulatory signaling pathways, including distinct receptors. In response to acute inflammation, these endogenous mediators blunt the inflammatory response by inhibiting aberrant neutrophil trafficking and activation, stimulating efferocytosis of apoptotic neutrophils and promoting anti-angiogenic, anti-fibrotic, and anti-infective actions. In allergic immune responses, resolvins enlist NK cells to facilitate clearance of activated T cells and activate macrophages (in a non-phlogistic manner) for phagocytic removal of allergen deposited in inflamed lung. Rapidly formed during inflammatory responses, these autacoids are also rapidly inactivated by eicosanoid oxidoreductases. In the setting of chronic inflammatory lung disease, airway levels of omega-3 fatty acids and pro-resolving mediators are decreased. With no curative therapy currently available for asthma or several other chronic inflammatory diseases, the development of resolvin stable analogs is leading to exciting new potential therapeutic approaches in acute and adaptive chronic inflammation that emphasize these natural homeostatic pathways.

## Conflict of Interest Statement

Mediators (resolvins and protectins) used and evaluated in this study have been licensed by the Brigham and Women’s Hospital (BWH) to Resolvyx. Bruce D. Levy has an equity interest in Resolvyx and receives a share of licensing income through BWH.

## Abbreviations

ALX/FPR2, lipoxin A_4_ receptor/formyl peptide receptor 2; AT-RvD1, aspirin-triggered-resolvin D1 (7*S*,8*R*,17*R*-trihydroxy-docosa-4*Z*,9*E*,11*E*,13*Z*,15*E*,19*Z*-hexaenoic acid); AT-RvD2, aspirin-triggered-resolvin D2 (7*S*,16*R*,17*R*-trihydroxy-docosa-4*Z*,8*E*,10*Z*,12*E*,14*E*,19*Z*-hexaenoic acid); AT-RvD3, aspirin-triggered-resolvin D3 (4*S*,11*R*,17*R*-trihydroxy-docosa-5,7*E*,9*E*,13*Z*,15*E*,19*Z*-hexaenoic acid); AT-RvD4, aspirin-triggered-resolvin D4 (4*S*,5*R*,17*R*-trihydroxy-docosa-6*E*,8*E*,10*Z*,13*Z*,15*E*,19*Z*-hexaenoic acid); BALF, bronchoalveolar lavage fluid; BLT1, LTB_4_ receptor; CMKLR1, chemokine receptor-like 1; DHA, docosahexaenoic acid; EPA, eicosapentaenoic acid; LC-UV-MS/MS, liquid chromatography-ultraviolet spectrometry-tandem mass spectrometry; LOX, lipoxygenase; LTB_4_, leukotriene B_4_ (5*S*,12*R*-dihydroxy-eicosa-6*Z*,8*E*,10*E*,14*Z*-tetraenoic acid); LXA_4_, lipoxin A_4_ (5*S*,6*R*,15*S*-trihydroxy-eicosa-7*E*,9*E*,11*Z*,13*E*-tetraenoic acid); PD1, protectin D1 (10*R*,17*S*-dihydroxy-docosa-4*Z*,7*Z*,11*E*,13*E*,15*Z*,19*Z*-hexaenoic acid); RvD1, resolvin D1 (7*S*,8*R*,17*S*-trihydroxy-4*Z*,9*E*,11*E*,13*Z*,15*E*,19*Z*-docosahexaenoic acid); RvD2, resolvin D2 (7*S*,16*R*,17*S*-trihydroxy-docosa-4*Z*,8*E*,10*Z*,12*E*,14*E*,19*Z*-hexaenoic acid); RvD3, resolvin D3 (4*S*,11,17*S*-trihydroxy-5*E*,7*E*,9*E*,13*Z*,15*E*,19*Z*-docosahexaenoic acid); RvD4, resolvin D4 (4*S*,5,17*S*-trihydroxy-6*E*,8*E*,10*Z*,13*Z*,15*E*,19*Z*-docosahexaenoic acid); RvE1, resolvin E1 (5*S*,12*R*,18*R*-trihydroxy-6*Z*,8*E*,10*E*,14*Z*,16*E*-eicosapentaenoic acid); RvE2, resolvin E2 (5*S*,18*R*-dihydroxy-8*Z*,11*Z*,14*Z*,16*E*-eicosapentaenoic acid; SAA, serum amyloid A; TGF-β, transforming growth factor-beta.
